# Identification of a Single-Nucleotide Insertion in the Promoter Region Affecting the *sodC* Promoter Activity in *Brucella neotomae*

**DOI:** 10.1371/journal.pone.0014112

**Published:** 2010-11-24

**Authors:** Dina A. Moustafa, Neeta Jain, Nammalwar Sriranganathan, Ramesh Vemulapalli

**Affiliations:** 1 Department of Comparative Pathobiology, Purdue University, West Lafayette, Indiana, United States of America; 2 Department of Biomedical Sciences and Pathobiology, Virginia Tech, Blacksburg, Virginia, United States of America; Instituto Butantan, Brazil

## Abstract

*Brucella neotomae* is not known to be associated with clinical disease in any host species. Previous research suggested that *B. neotomae* might not express detectable levels of Cu/Zn superoxide dismutase (SOD), a periplasmic enzyme known to be involved in protecting *Brucella* from oxidative bactericidal effects of host phagocytes. This study was undertaken to investigate the genetic basis for the disparity in SOD expression in *B. neotomae*. Our Western blot and SOD enzyme assay analyses indicated that *B. neotomae* does express SOD, but at a substantially reduced level. Nucleotide sequence analysis of region upstream to the *sodC* gene identified a single-nucleotide insertion in the potential promoter region. The same single-nucleotide insertion was also detected in the *sodC* promoter of *B. suis* strain Thomsen, belonging to biovar 2 in which SOD expression was undetectable previously. Examination of the *sodC* promoter activities using translational fusion constructs with *E. coli* β-galactosidase demonstrated that the *B. neotomae* and *B. suis* biovar 2 promoters were very weak in driving gene expression. Site-directed mutation studies indicated that the insertion of A in the *B. neotomae sodC* promoter reduced the promoter activity. Increasing the level of SOD expression in *B. neotomae* through complementation with *B. abortus sodC* gene did not alter the bacterial survival in J774A.1 macrophage-like cells and in tissues of BALB/c and C57BL/6 mice. These results for the first time demonstrate the occurrence of a single-nucleotide polymorphism affecting promoter function and gene expression in *Brucella*.

## Introduction

The genus *Brucella* consists of small, non-motile, non-spore forming, gram-negative, facultatively intracellular bacteria capable of infecting a variety of mammals. Infection with *Brucella* in animals leads to reproductive failure with abortions and infertility in females, and in some cases, orchitis in males [1], [2]. Brucellosis is a zoonotic disease, and humans usually acquire the infection by consuming contaminated dairy or meat products or by coming in contact with the infected animal tissues and secretions [1]. There are six well-recognized species of *Brucella* that show a marked host preference – *B. abortus* (cattle), *B. melitensis* (goat and sheep), *B. suis* (pig), *B. canis* (dog), *B. ovis* (sheep, especially ram), and *B. neotomae* (wood rat). In addition, isolates of *B. abortus*, *B. melitensis*, and *B. suis* are subdivided into biovars (or biotypes) based on the differences in their biological characteristics. Interestingly, *B. suis* biovar 2 is known to be incapable of causing significant clinical disease in pigs as well as in humans [3]. In the past few years, *Brucella* has been recovered from several marine mammals, including cetaceans (dolphin, whale and porpoise) and pinnipeds (seals and otters); these marine isolates belong to two potential new species, *B. pinnipedialis* and *B.ceti* [4]. Very recently, a new species of *Brucella*, *B. microti*, was isolated from wild common voles suffering from a systemic disease [5], [6].

The virulence of *Brucella* can be attributed to the ability of these bacteria to escape the host defense mechanisms and survive and replicate within the host cells. Virulent *Brucella* organisms are capable of invading and replicating in professional phagocytes, such as macrophages, as well as several types of non-phagocytic cells [7]. The ability of *Brucella* to maintain long-term residence in macrophages is the basis for establishing and maintaining chronic infections, a hallmark of brucellosis. Adaptation to intracellular survival within macrophages necessitates *Brucella* to resist the bactericidal action of reactive oxygen intermediates generated by the host cells. One of the mechanisms some bacteria employ to resist the oxidative killing by host macrophages is the production of periplasmic Cu/Zn-cofactored superoxide dismutase (SOD) enzyme that catalyzes the dismutation of superoxide (O_2_^−^) to hydrogen peroxide [8], which can be subsequently detoxified by the enzymatic action of catalases and peroxidases. In *Brucella spp.*, SOD is a periplasmic protein that is encoded by the *sodC* gene [9], [10]. Studies with *B. abortus* indicated that SOD plays a significant role in setup and maintenance of persistent infections in murine brucellosis models [11], [12].

*B. neotomae* was first isolated from pooled tissue suspensions of lung, spleen, liver and kidney of desert wood rats collected in the Great Salk Lake Desert in Utah [13]. In contrast to other *Brucella spp.*, there is no documented evidence indicating the pathogenic potential of *B. neotomae* in any host species. Though not much further research has been done on *B. neotomae*, it is generally considered to be a less virulent *Brucella* species. Limited animal studies conducted immediately after the initial isolation indicated that *B. neotomae* can infect mice, pigs, guinea pigs, and wood rats but does not cause any apparent clinical disease [13]–[15]. Perhaps because of its restricted host range and/or low virulence characteristics, *B. neotomae* has never been isolated from any other animal species. No disease in human has been attributed to *B. neotomae*.

Bricker *et al.* (1990) previously demonstrated that, of all the classical *Brucella* species and biovars examined, only *B. neotomae* and a *B. suis* biovar 2 isolate could not express detectable levels of SOD. The goal of this study was to determine the genetic basis for the lack of detectable SOD expression in *B. neotomae*. In this paper, we demonstrate that *B. neotomae* does express SOD, but at a substantially reduced level. Our studies discovered the presence of a single-nucleotide insertion in the promoter region of *sodC* gene as the cause for the reduced activity of this promoter in *B. neotomae* and *B. suis* biovar 2. Furthermore, we demonstrate that increasing the level of SOD expression in *B. neotomae* through complementation does not alter its persistence profile in mouse tissues.

## Materials and Methods

### Ethics statement

Some experiments described in this manuscript were performed in mice. The animal experiments were approved by Purdue Animal Care and Use Committee (PACUC No. 01-076-07).

### Bacterial strains

*B. neotomae* strain 5K33 was purchased from American Type Culture Collection. *B. abortus* strain RB51 was available in our laboratory. *B. abortus* virulent strain 2308 was from culture collection at Virginia Tech. *E. coli* DH5α was purchased from Invitrogen. All of the bacteria were grown in tryptic soy broth (TSB) or tryptic soy agar (TSA) at 37°C. Bacteria containing plasmids were grown in presence of appropriate antibiotics at 30- or 100-µg/ml concentrations of chloramphenicol and ampicillin, respectively. Colony forming units (CFU) of *B. neotomae* containing the recombinant plasmids were determined by plating the 10-fold serial dilutions of the cultures on TSA with and without appropriate antibiotics, to ascertain the stable maintenance of the plasmids in the recombinant bacteria.

### Complementation of *B. neotomae* with functional *sodC* gene

A 1.1 kb fragment containing the *B. abortus sodC* gene and its promoter sequence was excised from pBS/SOD [16] with *Cla*I restriction enzyme digestion and subcloned into pBBR4MCS to generate pBB4SOD. *B. neotomae* was transformed with pBB4SOD by electroporation following the previously described procedures [17]. *B. neotomae* containing the plasmid pBB4SOD (*B. neotomae*/pBB4SOD) was selected by plating the transformed bacteria on TSA plates containing ampicillin. The expression of SOD in the complemented *B. neotomae* was determined by SDS-PAGE and Western blotting.

### SDS-PAGE and Western blotting

For determining the expression of SOD in *B. neotomae* and in the complemented strain, SDS-PAGE and Western blot analyses were performed as described previously [18], [19]. The antigen extract prepared from *B. abortus* strain RB51 was used for the positive control for SOD expression. The amount of antigen loaded in each lane of the gel corresponded to total antigens of 10^7^ or 10^9^ CFU of *Brucella* harvested during late log phase of growth curve. For Western blotting, goat anti-*B. abortus* SOD sera was used as the primary antibody [20]. The membranes were developed with rabbit anti-goat IgG conjugated with horseradish peroxidase and a colorimetric substrate (KPL Inc., Gaithersburg, Maryland).

### Periplasmic extracts and SOD enzyme assay

Selective release of periplasmic contents of *B. neotomae*, *B. neotomae*/pBB4SOD, and *B. abortus* RB51 was performed as per the previously described procedure [21]. Briefly, bacteria harvested during different growth phases were pelleted down by centrifugation and resuspended in Tris-Hcl buffer, pH 7.5, at 100 µl per 10 mg of pellet wet weight. An equal volume of 0.2M Tris, pH7.5, containing 1M sucrose and 0.5% zwittergent 316 (Calbiochem) was added. Lysozyme was added to the suspension to achieve a final concentration of 100 µg/ml. Bacterial cells were exposed to a mild osmotic shock by adding an equal volume of distilled water. The suspensions were shaken for 2 hours at room temperature and then centrifuged at 8,000× g for 30 min and the supernatants were used for measuring SOD enzyme activity. The protein concentration of the supernatant was determined using a Bio-Rad protein assay kit.

Quantitative determination of SOD activity was achieved by a colorimetric microtiter plate method using a commercially available SOD assay kit (Dojindo Molecular Technologies, Inc.). SOD activity was assayed at 450 nm as the inhibition of the reduction of the tetrazolium salt 2-(4-iodophenyl) 3-4-(nitrophenyl)-5(2,4-disulfophenyl)-2H tetrazolium (WST-1) by superoxide produced by xanthine oxidase. Commercially available *E. coli* SOD (Sigma-Aldrich) was used to construct standard curves. Enzyme activity, defined as percent inhibition of WST-1 reduction, was determined as {[(reagent control *v_o_*−buffer blank *v_o_*)−(sample *v_o_*−sample blank *v_o_*)]/(reagent control *v_o_*−buffer blank *v_o_*)}×100 for both samples and standards. SOD activity per well was determined from the standard curve. With each sample, the SOD assay was performed twice, each time in duplicates, and the data were reported as specific activity (SOD units/mg of protein).

### PCR amplification and nucleotide sequence analysis

The coding sequences and the upstream region of the *sodC* gene from the genomic DNA of *B. neotomae*, *B. suis* biovars 2 (strain Thomsen) and 4 (strain 40) were amplified via PCR using specific primer-pairs - SOD-RBS-F (5′-GGGAATGGCCTTACGGTT-3′) and SOD-RBS-R (5′-TTATTCGATCACGCCGCA-3′) for coding region, and SOD-upstream-F (5′-CGCAGCCACCCGTTCATGTT-3′) and SOD-upstream-R (5′-GTCGGCAGCGCCTCATAC-3′) for upstream region. The genomic DNA of *B. suis* strains was previously obtained from National Animal Disease Center, Ames, Iowa [22]. The genomic DNA of *B. neotomae* and *B. abortus* RB51 was extracted using DNeasy Tissue kit (Qiagen) following the manufacturer's recommended procedure for DNA isolation from bacteria. Twenty ng of template DNA,10 pmol of each of the specific primer-pairs and reagents from NovaTaq PCR Kit PLUS (Novagen) were used to prepare PCR mixtures in a total volume of 50 µl. The PCR amplifications were performed using a thermocycler (iCyler, Bio-Rad Laboratories) with the following conditions: 95°C for 4 min followed by 30 cycles that each included 30 sec of denaturation at 95°C, 30 sec of annealing at 58°C, and 30 sec of extension at 72°C. The amplified PCR products were cloned into the pCR2.1 vector, using the TA cloning system (Invitrogen). The nucleotide sequences of both strands of the cloned fragments from 3 independent clones were determined by DNA sequencing using M13 forward and reverse primers at Purdue Genomics Core Facility. The nucleotide sequences were analyzed using LaserGene sequence analysis software (DNASTAR, Inc.). The *B. neotomae* nucleotide sequences were submitted to databases at GenBank (Accession number EU056817).

### RNA extraction and RT-PCR

Total RNA was extracted from *B. neotomae* and *B. abortus* RB51 using RNeasy Mini Kit (Qiagen) according to the manufacturer's protocol for DNA-free RNA isolation from bacteria. Bacteria harvested during late log phase of growth curve were used for the RNA extraction. The extracted RNA concentration was determined using Quant-iT RiboGreen RNA reagent and kit (Molecular Probes). Using SuperScript™ One-step RT-PCR kit (Invitrogen), RT-PCR to detect *sodC* mRNA was performed with 40 ng of the extracted RNA and primers SOD-RBS-F and SOD-upstream-R. cDNA synthesis and PCR amplification were performed in the same tube with the following thermocycler conditions: cDNA synthesis at 45°C for 30 min, inactivation of reverse-transcriptase enzyme at 95°C for 10 min, amplification of cDNA sequences with 40 cycles of 95°C for 1 min, 58°C for 1 min, and 72°C for 1 min. As a control to rule out DNA contamination of the extracted RNA, a set of reaction tubes were subjected to direct PCR amplification by omitting the reverse transcription step (45°C for 30 min) of above mentioned thermocycler conditions. Following the PCR amplification, 12 µl of the reaction mixtures were separated on a 2% agarose gel electrophoresis and stained with ethidium bromide to visualize the amplified products.

### 5′ RACE reaction

Amplification of the *sodC* cDNA 5′ end was performed using the 5′ RACE System Version 2.0 for rapid amplification of cDNA ends kit (Invitrogen) as per the manufacturer's suggested protocol. Briefly, the first strand cDNA was synthesized from the bacterial RNA using the gene specific primer 1 (GSP1) SOD-Pext-R (5′-GGGCTTCGGAAATGACCACG-3′) and SuperScript II under the following conditions: denaturation of the RNA at 70°C for 10 min, followed by cDNA synthesis at 42°C for 50 min. The template RNA was then removed by treating the reaction mixture with RNase mix. Unincorporated dNTPs and GSP1 were separated from the cDNA by SNAP column purification. A homopolymeric tail was added to the 3′ end of the cDNA using terminal deoxynucleotidyl transferase (TdT) and dCTP at 37°C for 10 min. Following inactivation of TdT at 65°C for 10 min, the tailed cDNA was PCR amplified using gene specific primer 2 (GSP2), SOD-upstream R, and abridged-anchor primer with the following thermocycler conditions: initial denaturation at 94°C for 1 min, 35 cycles of 94°C for 1 min, 55°C for 1 min, and 72°C for 1 min, and a final extension step at 72°C for 7 min. In order to generate enough specific product, a nested PCR amplification was performed using GSP2 and abridged universal anchor primer using the previous thermocycler conditions. The PCR products were separated by electrophoresis on a 2% agarose gel, eluted using a QIAEX II gel extraction kit (Qiagen) and cloned into pGEM-T vector (Promega). The nucleotide sequences of the cloned fragments were determined by DNA sequencing using M13 reverse primer.

### Construction of plasmids for determining the promoter strength

The strength of *sodC* promoters from *B. neotomae*, *B. abortus*, and *B. suis* biovars 2 and 4 was determined by generating translational fusion with *E. coli* β-galactosidase and then measuring the enzymatic activity of the expressed β-galactosidase in *B. neotomae* transformed with the plasmids. For this, a 138 bp fragment previously determined to contain the *sodC* promoter region, the ribosomal binding site and the start codon [16] was amplified from the genomic DNA of corresponding *Brucella* via PCR using a specifically designed primer-pair, SOD-pro-F (5′- AAGTCGACATCGATAATTTCGGGGTGGAGA-3′) and SOD-pro-R (5′-AACTGCAGATCATCACTTCTCCTGAATATAGTTAGA-3′). A restriction site was added at the 5′ end of the each primer (*Sal*I in SOD-pro-F and *Pst*I in SOD-pro-R) to facilitate directional cloning into plasmid pNSGroE/lacZ [23]. The amplified DNA fragments were first cloned into pCR2.1 vector and sequenced to ascertain the integrity of the nucleotide sequences. The inserts were then excised from the pCR2.1 vector by digesting with *SalI* and *Pst*I restriction enzymes and cloned into plasmid pNSGroE/lacZ digested with the same enzymes, resulting in the replacement of the *groE* promoter with the cloned *sodC* promoter. In the resulting recombinant plasmids, the start codon of the *sodC* gene was in-frame with the coding sequences of the *lacZ* gene, which was again confirmed by nucleotide sequencing using the SOD-pro-F primer. The resulting plasmids were designated pBnSODpro/lacZ, pBaSODpro/lacZ, pBs2SODpro/lacZ, and pBs4SODpro/lacZ. *B. neotomae* was transformed with these plasmids and the expression of β-galactosidase in the recombinant bacteria was measured.

### Site-directed mutagenesis

To alter specific nucleotide(s) in the predicted promoter region of *B. neotomae sodC* gene, PCR amplification was performed with a common reverse primer (SOD-pro-R) and one of the three forward primers, B.n-F-m1 (5′- TTTAGCAATTGCTGTAACTA**T**TTGTTTTAATACAGTA-3′), B.n-F-m2 (5′-TTTAGCAATTGCTGTAACTAATT**T**TTTTAATACAGTA-3′), or B.n-F-2m1 (5′- TTTAGCAATTGCTGTAACTA**T**TT**T**TTTTAATACAGTA-3′) containing the specific mutations. Similarly, two single-nucleotide mutations were generated in the potential promoter region of *B. suis* biovar 4 *sodC* gene using the forward primers B.s4-F-m1 (5′- TTTAGCAATTGCTGTAACTA**A**TGTTTTAATACAGTA-3′) and B.s4-F-m2 (5′-TTTAGCAATTGCTGTAACTATTG**G**TTTAATACAGTA-3′). The 5′ ends of the forward primers with the specific mutations encompass the sequence containing the site for *Mfe*I restriction enzyme. The amplified 110 bp DNA product was digested with *Mfe*I and *Pst*I restriction enzymes and cloned into plasmid pBnSODpro/lacZ previously digested with the same enzymes, resulting in the replacement of the wild type sequences with that containing specific mutated nucleotide(s) which was confirmed by nucleotide sequencing. The resulting plasmids were designated pBnSODm1/lacZ, pBnSODm2/lacZ, pBnSOD2m1/lacZ, pBs4SODm1/lacZ and pBs4SODm2/lacZ. *B. neotomae* was transformed with these plasmids and the expression of β-galactosidase in the recombinant bacteria was measured. *B. abortus* virulent strain 2308 and vaccine strain RB51 were also transformed with plasmids containing the *B. neotomae sodC* promoter variants and the β-galactosidase expression in the corresponding recombinant bacteria was measured.

### *β-Galactosidase* enzyme assay

β-Galactosidase was assayed in *B. neotomae*, B. *abortus* 2308 and *B. abortus* RB51 by the methods described previously [24]. The enzyme activity was calculated in Miller units with the following formula: (OD_420_×1000/*t*×*v*×OD_600_), where the OD_420_ is the optical density at 420 nm, *t* is the incubation time in minutes, *v* is the volume of culture used in milliliters, and OD_600_ is the optical density of the bacterial culture used for enzyme assay. The assays were performed in triplicate with bacteria grown from 3 separate colonies of each strain.

### Survival and replication of *B. neotomae* and *B. neotomae*/pBB4SOD in J744A.1 cells

The intracellular growth characteristics of *B. neotomae* and *B. neotomae* complemented with the functional *sodC* gene (*B. neotomae* harboring pBB4SOD) was determined in J774A.1 macrophage-like cells using the method previously described [25]. Briefly, J774A.1 cells were cultured overnight in 6-well plates with antibiotic-free medium and infected with 10^8^ CFU of *B. neotomae* or *B. neotomae*/pBB4SOD as described above for intracellular β-galactosidase expression analysis. For each bacterial strain, the assay was performed in triplicate by infecting cells in 3 wells. At different time-points, the infected monolayers were washed three times with antibiotic free media, and the cells were lysed using 0.1% deoxychloate solution. The CFU of bacteria released from the cells were determined by plating 10-fold serial dilution of the lysates on TSA; for *B. neotomae*/pBB4SOD infected cells, the CFU were also determined by plating the diluted lysates on TSA with ampicillin.

### Mice Experiments

The in vivo growth characteristics of *B. neotomae* and *B. neotomae* complemented with pBB4SOD were determined in mice. Four-week old, female BALB/c and C57BL/6 mice were purchased from Harlan. Mice were given 1 week of rest before the experiments were started. Each strain of mice were divided into two groups, and mice in one group were injected intraperitoneally each with 3×10^6^ CFU of *B. neotomae* and mice in the other group were similarly injected with 1.8×10^6^ CFU of *B. neotomae* harboring pBB4SOD. The actual CFU injected was determined retrospectively by plating the inoculum on TSA plates. At specific time-points after infection, three mice from each group were euthanized, their spleens and livers were removed and the numbers of CFU were determined by plating 10-fold serial dilutions of tissue homogenates on TSA, and also on TSA with ampicillin in case of *B.neotomae*/pBB4SOD inoculated mice.

### Statistical analyses

One-way ANOVA with Tukey post test was performed using GraphPad Prism (GraphPad Software, San Diengo, CA, USA) to analyze the enzymatic activities of SOD and β-galactosidase. Student's *t* test was used to analyze the intracellular bacterial counts and the bacterial CFU in the spleens of mice.

## Results

### Expression of SOD in *B. neotomae*

**Figure 1:**
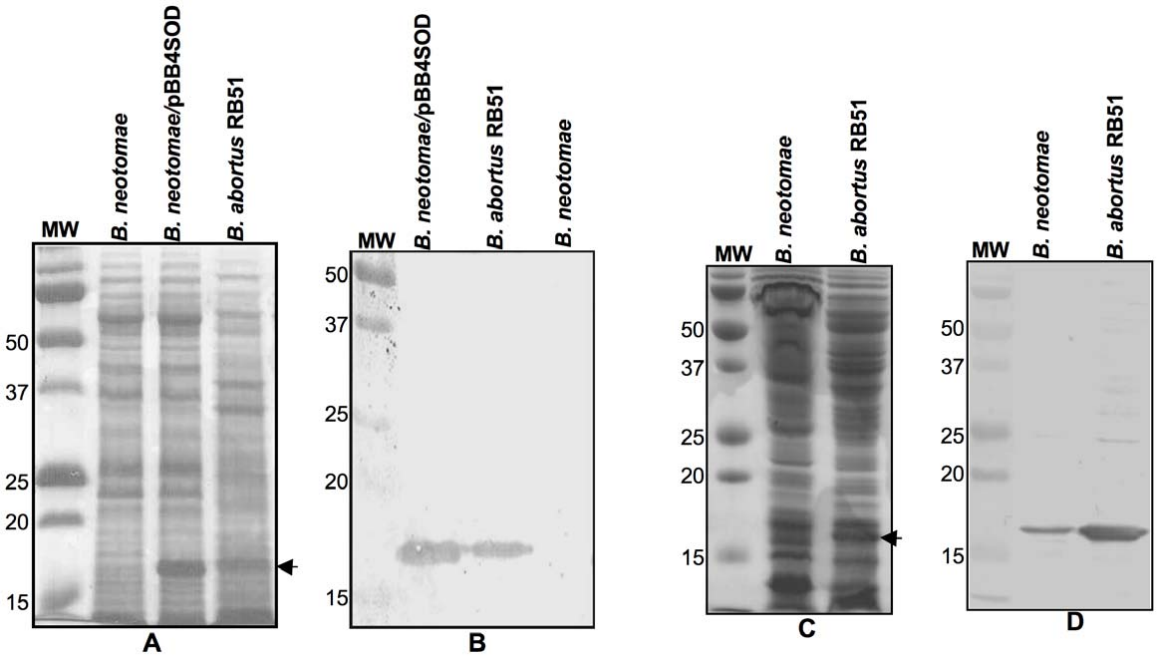
Detection of SOD expression in *B. neotomae*, *B. neotomae*/pBB4SOD, and *B. abortus* RB51 by SDS-PAGE (A and C) and Western blot analysis (B and D). Total antigens of 10^7^ (A and B) or 10^9^ (C and D) CFU of the indicated bacteria were loaded on each lane. The SDS-PAGE gels were stained with Coomassie brilliant blue G (A and C). For Western blot analysis (B and D), goat anti-*Brucella* SOD serum was used as the primary antibody for reacting with the antigens. In all panels, lanes marked MW contain molecular weight markers and the numbers at the left indicate approximate molecular masses in kilodaltons. Arrows in panels A and C indicate the SOD protein.

Low levels of SOD expression was detected in *B. neotomae* strain 5K33 by Western blot analysis using specific polyclonal antibodies. As shown in Fig. 1A and B, no SOD was detected in the total antigen extract from 10^7^ CFU of *B. neotomae*, while considerable amount of SOD was present in the antigen extracts from the same number of *B. neotomae* containing plasmid pBB4SOD. When the Western blot analysis was performed using the total antigen extracts obtained from 10^9^ CFU of *B. neotomae*, a thin protein band with size corresponding to the SOD consistently reacted with the SOD-specific antibodies (Fig. 1C and D). As expected, SOD was present in the antigen extracts of both 10^7^ and 10^9^ CFU of *B. abortus* RB51.

### SOD activity in periplasmic extracts

**Figure 2:**
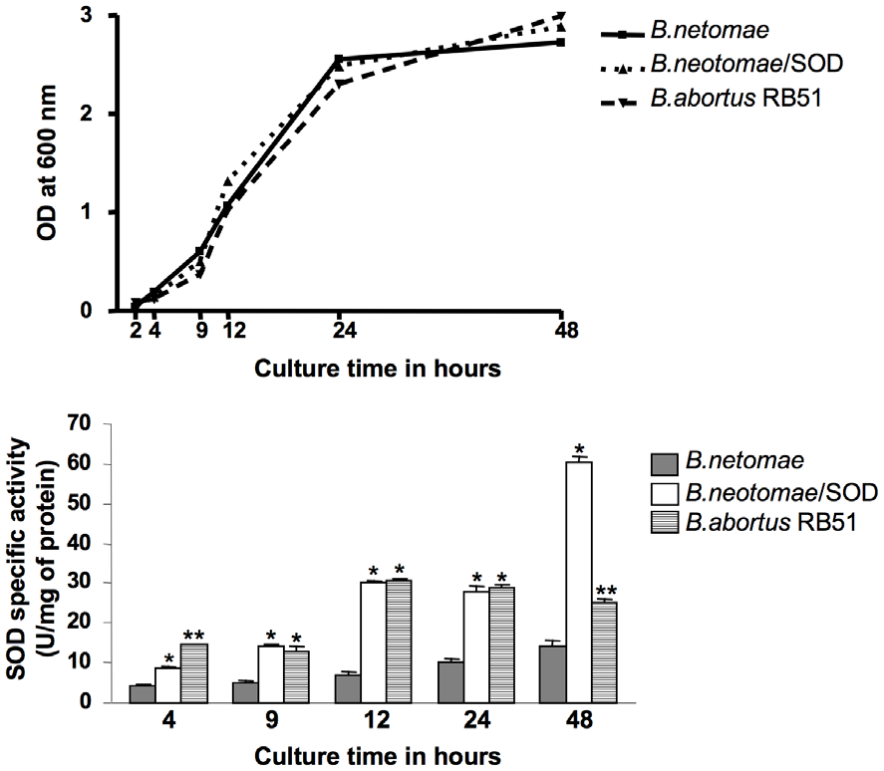
Specific activity of SOD enzyme in the periplasmic extracts of *B. neotomae*, *B. neotomae*/pBB4SOD, and *B. abortus* RB51 at different growth stages. The growth curves of the bacteria are shown in the top panel. The specific activities of SOD enzyme are shown in the bottom panel. For each *Brucella*, overnight culture was used at 1∶200 dilution to inoculate fresh 100 ml media and at different time intervals, the OD_600_ was measured and an aliquot of the culture was taken for preparing periplasmic extracts. With extracts of each time-point, the SOD assay was performed twice, each time in duplicates, and the results are shown as the mean ± standard deviation of specific activity (units/mg of protein). At each time point, the groups with one or two asterisks were significantly different from the *B. neotomae* group (*P*<0.001). At 4 and 48 hours time-points, there were significant differences between the groups with different number of asterisks (*P*<0.001).

As shown in Fig. 2, SOD activity was detected in *B. neotomae*, *B. neotomae*/pBB4SOD and *B. abortus* RB51 at all time-points tested during the 48-hour culture period. The maximum enzyme activity was detected in *B. neotomae* during late log phase (24 hours) and stationary phase (48 hours). However, at all time-points tested, the SOD specific activity in *B. neotomae* was lower than that of *B. abortus* RB51 and *B. neotomae*/pBB4SOD.

### Sequence analysis of *B. neotomae sodC* gene

**Figure 3:**
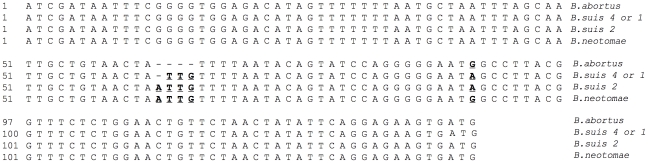
Multiple alignment of nucleotide sequences of the 5′ flanking region of *sodC* start codon from *B. abortus*, *B. suis* biovars 4 (strain 40) or 1 (strain 1330) and 2 (strain Thomsen), and *B. neotomae*. Alignment was performed with the Clustal method of the MegAlign program of LaserGene software. Gaps are indicated by dashes. The nucleotides differing between *B. suis* and *B. abortus* or *B. neotomae* are in bold and underlined. Note: *B. melitensis* nucleotide sequences are identical to those of *B. abortus* shown above.

In order to verify if the low level of SOD expression in *B. neotomae* is because of mutations within the coding or the upstream regions of *sodC* gene, we first performed PCR amplification using *B. neotomae* genomic DNA as template and a primer-pair designed based on the known *sodC* gene sequence from *B. abortus*. The PCR amplification resulted in a 575 bp product, the expected size in the presence of a complete *sodC* gene. Nucleotide sequence of the amplified product showed 99.7% identity with the corresponding region from *B. suis* and 99.5% identity with that from *B. abortus* or *B. melitensis*. The *B. neotomae* sequence differed in 2 and 3 nucleotides from that of *B. suis* and *B. abortus* or *B. melitensis*, respectively. All the nucleotide differences were located within the open reading frame of the *sodC* gene and only one of the differed nucleotides caused a change in the deduced amino acid sequence, a F→V change at amino acid position 52 of the precursor polypeptide (data not shown). We then amplified a 350 bp region flanking the 5′ end of the start codon from the *B. neotomae* DNA. Nucleotide sequence analysis of this region revealed that *B. neotomae* differed in 2 nucleotides from that of the *B. suis* strain 1330 (biovar 1) sequence (GenBank accession no. NC_009504), one was a substitution of A with G and the other was an insertion of A (Fig. 3). Both the changes were within the 138 bp region upstream to the start codon that was previously shown to contain the *sodC* promoter element in *B. abortus* [16]. We also PCR amplified and sequenced the upstream region from strains belonging to *B. suis* biovars 2 (strain Thomsen) and 4 (strain 40). While the *B. suis* biovar 4 sequence was 100% identical with that of *B. suis* biovar 1, the sequence from *B. suis* biovar 2 differed in just 1 nucleotide, an insertion of A at the same location as that found in the *B. neotomae* sequence. Computer analysis of nucleotide sequences reported in the databases indicated 100% identity within the *sodC* upstream region between *B. abortus* and *B. melitensis*, and compared to *B. suis* biovars 1 and 4, these two bacteria contained 3 fewer nucleotides at the location where the nucleotide insertion was detected in *B. neotomae* and *B. suis* biovar 2 (Fig. 3). In addition, the nucleotide 48 upstream to the start codon was G in *B. abortus* and *B. melitensis*, but it was A in *B. suis*.

### Determining the 5′ end of *sodC* mRNA

**Figure 4:**
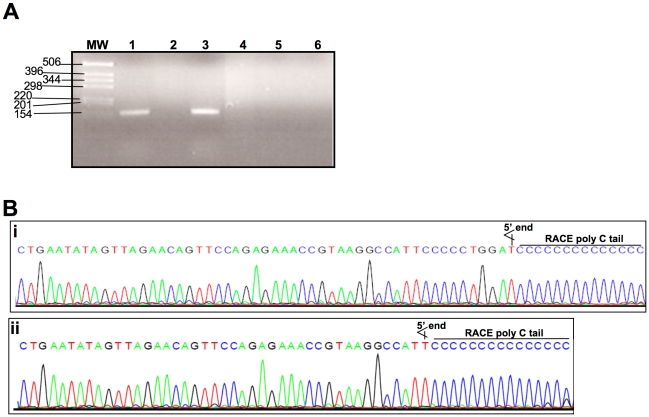
RT-PCR detection of *sodC* mRNA in *B. neotomae* and *B. abortus* RB51 (A) and identification of their 5′ ends by RACE analysis (B). A) Total RNA extracted from the bacterial cultures at late log phase was used as template in RT-PCR or direct PCR for amplification of a 5′ portion of *sodC* mRNA using a pair of specific primers. Post-amplification reaction mixtures of tubes containing RNA extracted from *B. neotomae* (lanes 1 and 2) or *B. abortus* RB51 (lanes 3 and 4) were separated on a 2% agarose gel by electrophoresis and stained with ethidium bromide to visualize the DNA fragments. Lanes 1 and 3, RT-PCR reaction mixtures. Lanes 2 and 4, PCR reaction mixtures. Lanes 5 and 6, reaction mixtures from no-template-controls of RT-PCR and PCR reactions, respectively. Lane marked MW contains DNA molecular size marker, and the numbers at the left indicate molecular size in base-pairs. B) Representative nucleotide sequencing chromatograms showing the location of 5′ ends of the cloned *sodC* cDNAs from *B. abortus* RB51 (i & ii) and *B. neotomae* (ii).

RT-PCR amplification resulted in specific amplification of 150 bp cDNA product from RNA extracted from both *B. neotomae* and *B. abortus* RB51 (Fig. 4A). No amplified products were detected when reverse-transcription step was omitted, ruling out the genomic DNA contamination as the source for the specific amplification obtained with RT-PCR (Fig. 4A). Nested PCR products of 5′ RACE reaction from both *B. neotomae* and *B. abortus* RB51 contained a ∼150 bp DNA fragment. The PCR products were cloned in pGEM-T vector and recombinant plasmids from 6 independent colonies for each *Brucella* species were used for sequencing the cloned products. For *B. abortus* RB51, two of the recombinant plasmids identified ‘A’ at 61 nucleotides upstream to the start codon as the 5′ end of the cDNA (Fig. 4Bi). In all 6 of the *B. neotomae* and the remain 4 of the *B. abortus* RB51 recombinant plasmids, ‘A’ at 51 nucleotides upstream to the start codon was found to be the 5′ end of the cDNA (Fig. 4Bii); however, because of the C-tailing of the RACE reaction, we cannot rule out the possibility of anyone of the five ‘G’ nucleotides present immediately upstream to the A could be the actual 5′ end. In either case, the findings indicated that the 5′ end of the *sodC* gene was downstream to the region showing nucleotide sequence variability among certain *Brucella* species or biovars.

### Reduced activity of *B. neotomae sodC* promoter

**Figure 5:**
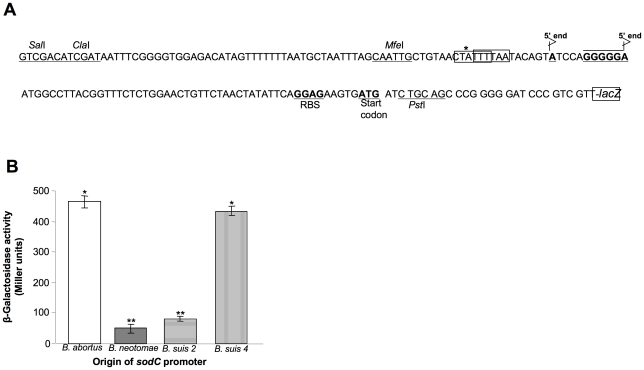
Evaluation of strength of *sodC* promoters from *B. abortus*, *B. neotomae*, *B. suis* biovars 2 (strain Thomsen) and 4 (strain 40). A) Nucleotide sequence features of the promoter-containing 5′ flanking region of *B. abortus sodC* gene as cloned in pBaSODpro/lacZ. The *sodC* start codon, ribosomal binding site (RBS), and 5′ ends of cDNA are indicated in bold. The asterisk indicates the site of nucleotide insertion polymorphism with *B. neotoame* and *B. suis* biovars. The two potential −10 sequences are boxed (see Discussion). B) Expression levels of β-galactosidase in late log phase cultures of *B. neotomae* cultures harboring plasmids with *lacZ* gene cloned under the control of *sodC* promoters obtained from the indicated *Brucella* spp. For each promoter construct, the assays were performed using 3 separate colony cultures. Results are shown as the mean ± standard deviation of Miller units. Means with the same number of asterisks were not significantly different from each other (*P*>0.05). There were significant differences between means with different number of asterisks (*P*<0.001).

In order to verify if the identified nucleotide insertion in the *sodC* upstream region of *B. neotomae* and *B. suis* biovar 2 affected the promoter activity, we assessed the strength of the *sodC* promoters by constructing translational fusions with *E. coli* β-galactosidase enzyme by generating plasmids pBnSODpro/laZ, pBaSODpro/lacZ, pBs2SODpro/lacZ, and pBs4SODpro/lacZ (Fig. 5A). The level of β-galactosidase expression in *B. neotomae* harboring these plasmids was determined by measuring the enzyme activity during late log phase (24 hour cultures). The expression of β-galactosidase under the *B. neotomae* and *B. suis* biovar 2 promoters was considerably lower than the expression under *B. abortus* and *B. suis* biovar 4 promoters (Fig. 5B).

**Figure 6:**
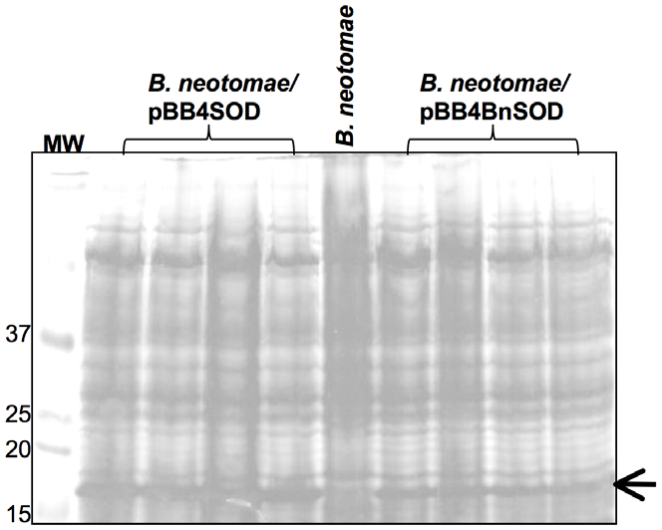
Detection of SOD overexpression in *B. neotomae*/pBB4SOD and *B. neotomae*/pBB4BnSOD by SDS-PAGE. Total antigens of the indicated bacteria were prepared from different individual recombinant colonies and loaded on each lane. The gels were stained with Coomassie brilliant blue G. The lane marked MW contain molecular weight markers and the numbers at the left indicate approximate molecular masses in kilodaltons. Arrow indicates the SOD protein.

To examine the effect of promoter activity on SodC expression, the *sodC* gene along with its own promoter was amplified from *B. netomae* and cloned into pBBR4MCS plasmid to obtain pBB4/BnSOD. *B. neotomae* was transformed with pBB4/BnSOD and the resulting recombinant strain was designated *B. neotomae*/pBB4BnSOD. SDS-PAGE analysis indicated that the increased expression of SOD in *B. neotomae*/pBB4BnSOD compared to that of *B. neotome* (Fig. 6), which could be attributed to the increased gene copies because of the number of plasmid copies (19). However, the level of SOD overexpression in *B. neotomae*/pBB4BnSOD was clearly lower than that observed in *B. neotomae*/pBB4SOD (Fig. 6).

### Determining the effect of single-nucleotide insertion on the promoter activity

**Figure 7:**
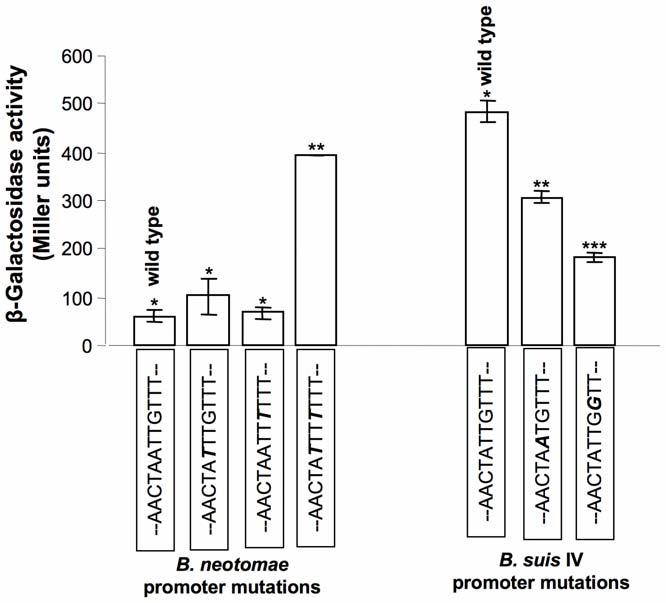
β-Galactosidase activity in *B. neotomae* transformed with plasmids containing *lacZ* gene under the control of wildtype and the indicated mutated *B. neotomae* and *B. suis* biovar 4 (strain 40) *sodC* promoters. For each promoter construct, the assays were performed using 3 separate colony cultures. Results are shown as the mean ± standard deviation of Miller units. In each panel, means with the same number of asterisks were not significantly different from each other (*P*>0.05), but there were significant differences between means with different number of asterisks (*P*<0.001).

The results of the above promoter strength assessment and 5′ RACE experiments indicated that the region with the single-nucleotide insertion in *B. neotomae* and *B. suis* biovar 2 was part of the potential *sodC* promoter. To verify if nucleotide sequences in this region could affect the *sodC* promoter activity, we generated 3 separate site-directed mutation constructs with plasmid pBnSODpro/lacZ to change the *B. neotomae* sequences from TAATTG to TATTTG (pBnSODm1/lacZ), TAATTT (pBnSODm2/lacZ), or TATTTT (pBnSOD2m1/lacZ). Similarly, we also generated 2 separate site-directed mutations in the *sodC* upstream sequences of *B. suis* biovar 4 to change the sequences from TATTGT to TAATGT (pBs4SODm1) or TATTGG (pBs4SODm2). Plasmids containing the original and the altered sequences were electroporated into *B. neotomae* and the level of β-galactosidase expression was determined using 3 separate colonies for each plasmid construct. As shown in Fig. 7, with the *B. neotomae* protomer, both the single-nucleotide mutations did not alter the promoter activity while the double-nucleotide mutation increased the promoter activity significantly but not to the same levels seen with the wild-type *sodC* promoter of *B. suis* biovar 4. With the *B. suis* biovar 4 promoter, both single-nucleotide mutations decreased the promoter activity.

**Figure 8:**
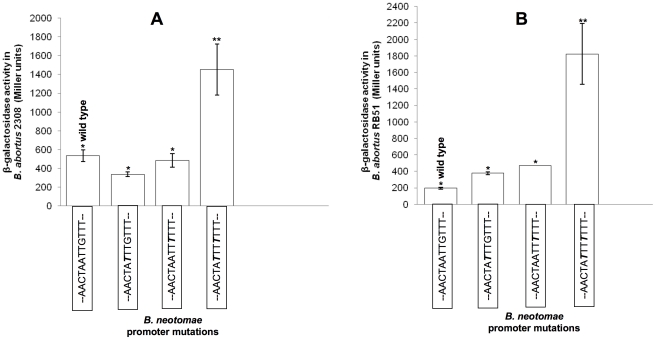
β-Galactosidase activity in *B. abortus* virulent strain 2308 (A) and *B. abortus* vaccine strain RB51 (B) transformed with plasmids containing *lacZ* gene under the control of wildtype and the indicated mutated *B. neotomae sodC* promoters. For each promoter construct, the assays were performed using 3 separate colony cultures. Results are shown as the mean ± standard deviation of Miller units. In each panel, means with the same number of asterisks were not significantly different from each other (*P*>0.05), but there were significant differences between means with different number of asterisks (*P*<0.001).

Activities of the *B. neotomae* wild-type and the mutated *sodC* promoters were also assessed in *B. abortus* virulent strain 2308 and vaccine strain RB51. The β-galactosidase expression analysis in *B. abortus* strains harboring the plasmid constructs showed that the original *B. neotomae sodC* promoter and its two single-nucleotide mutation variants were significantly less active than the variant with double-nucleotide mutation (Fig. 8).

### Intracellular replication of *B. neotomae* and *B. neotomae* expressing SOD in J774A.1 cells

**Figure 9:**
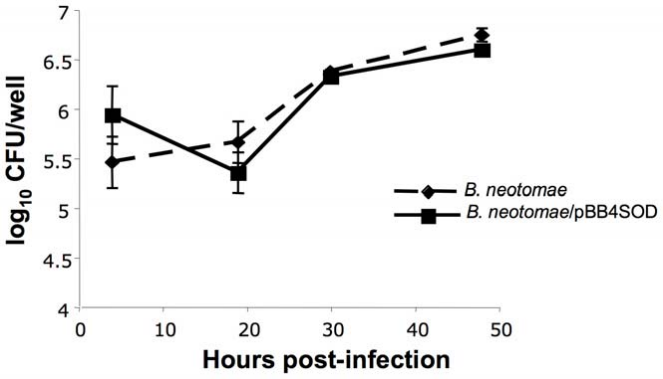
Intracellular survival and replication of *B. neotomae* and *B. neotomae*/pBB4 SOD in J774A.1 cells. Infection of J774A.1 cells was performed in triplicate as described in Materials and Methods, and for each time-point, results are shown as the mean ± standard deviation of log_10_ CFU per well. At each time-point there was no significant difference between the two groups (*P*>0.05).

We determined the intracellular growth kinetics of *B. neotomae* and *B. neotomae*/pBB4SOD by infecting J774A.1 cells and then determining the number of viable intracellular bacteria at different time-points. As shown in Fig. 9, no obvious differences were detected between the two bacterial strains in their ability to enter and replicate within the cells up to 48 hours.

### Infection and persistence profiles of *B. neotomae* and *B. neotomae* expressing SOD in mice

**Figure 10:**
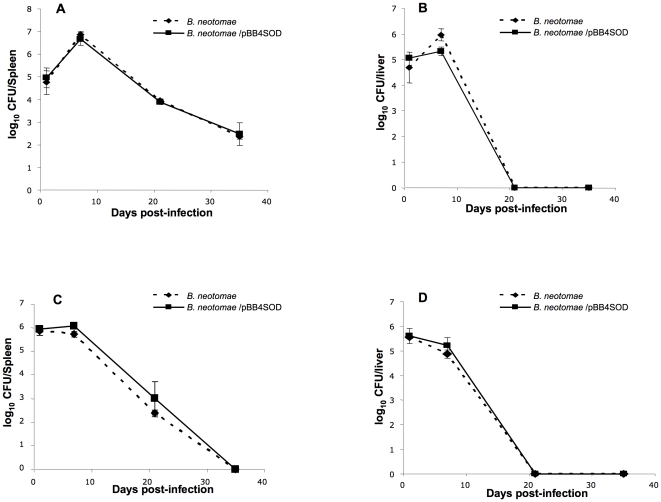
Colonization and persistence of *B. neotomae* and *B. neotomae*/pBB4SOD in spleens (A and C) and livers (B and D) of female, BALB/c (A and B) and C57BL/6 (C and D) mice. Mice were infected with the bacteria as described in Materials and Methods, and at each time-point, three mice from each group were euthanized and the bacterial burden in their spleens and livers was determined. Results are shown as the mean ± standard deviation of log_10_ CFU per organ. At each time-point there was no significant difference between the two groups (*P*>0.05).

BALB/c and C57BL/6 mice were infected with ∼10^6^ CFU of *B. neotomae* and *B. neotomae*/pBB4SOD, and the number of bacteria colonizing the spleens and livers was determined over time. In both strains of mice, no significant differences were observed in the bacterial counts between the *B. neotomae* and *B. neotomae*/pBB4SOD infected groups at all time-points tested. Based on the bacterial CFU recovered from the spleens and livers of infected BALB/c mice at 1 and 7 days post-infection, both *B. neotomae* and *B. neotomae*/pBB4SOD were able to establish infection and replicate in the tissues. However, at 3 weeks post-infection, there was a 3 log decrease in the bacterial burden in the spleens and no bacteria were recovered from the livers, indicating the inability of these bacteria to maintain a chronic infection (Fig. 10A & B). In case of C57BL/6 mice, *B. neotomae* and *B. neotomae*/pBB4SOD were able to establish an infection in the spleens and livers, but in contrast to BALB/c mice, no increase in the bacterial numbers was detected at day 7 post-infection. While the livers were free of any bacteria by week 3 post-infection, bacterial counts in the spleens decreased significantly at that time-point and by week 5 post-infection, the spleens of the mice were also free any bacteria (Fig. 10C & D).

## Discussion

Our Western blot and SOD enzyme assay analyses indicated that *B. neotomae* does express SOD, but at substantially low levels in comparison to *B. abortus* RB51. The reported preliminary data of Bricker *et al.* (1990) suggested no SOD expression in *B. neotomae*. Perhaps the amount of antigen and the concentration of the SOD-specific antibodies used by the previous researchers in their immunoblot analyses were not sufficient to detect the low levels of SOD expression in *B. neotomae*. The time of harvesting bacteria for antigen preparation could also determine the amount of SOD present. Our analysis of enzymatic activity indicated that the maximum expression of SOD in *B.neotomae* occurred during the late log and stationary phases, which is in agreement with the previous reports of SOD expression in bacteria increasing in a growth-phase-dependant manner and reaching the maximal level during stationary phase [26]. Regulation of SodC expression in *Brucella* is not clearly understood. Studies in *E. coli* indicate that upregulation of expression of certain genes, including *sodC*, during the stationary phase depends on the promoter recognition by the alternate sigma factor σ^S^ (RpoS) encoded by *rpoS* gene [27]. Expression of RpoS is in turn controlled by host factor-I encoded by *hfq* gene [28]. In *B. abortus* also SodC expression is *hfq* dependent [11]. However, analysis of genome sequences does not indicate that *Brucella* possesses an *rpoS*-like gene [29]. How the *hfq* gene product regulates *Brucella sodC* expression remains to be unraveled.

The only nucleotide change with potential to affect the expression of SOD in *B. neotomae* was found within the promoter containing region. The same nucleotide change, insertion of A in comparison with *B. suis* biovars 1 & 4, was also found in *B. suis* biovar 2, another *Brucella* previously shown not to express detectable levels of SOD [10]. The β-galactosidase expression results in *B. neotomae* indicated that the *B. suis* biovar 2 and *B. neotomae sodC* promoters were very weak in driving the gene expression compared to those from *B. abortus* and *B. suis* biovar 4. It should be noted that all the plasmid constructs tested in the present study were stably maintained in *B. neotomae*, as ascertained by the similar number of CFU obtained by plating the recombinant bacteria on TSA and TSA with appropriate antibiotics. Therefore, the difference in the β-galactosidase expression levels was not because of variation in plasmid stability.

Our 5′ RACE analysis showed that the 5′ end of *sodC* mRNA in *B. neotomae* was at 51–56 nucleotides upstream to the start codon. The *B. abortus* RB51 mRNA had two different 5′ ends, but one of it was identical to that of *B. neotomae*. These mRNA 5′ ends may represent the actual *sodC* transcription start sites, though the possibility that they could be products of post-transcriptional RNA processing or degradation cannot be ruled out. Identification of the 5′ end of the *sodC* mRNA supported the previous observations about the potential location of the promoter region [16]. It should be emphasized that only analysis based on experiments such as DNase I footprinting can confirmatively identify the promoter sequences. Our objective in carrying out the site-directed mutagenesis studies of the promoter region was to determine if nucleotides present at the region where a nucleotide insertion was detected in *B. neotomae* can affect *sodC* promoter function. Not much information is available on the sequence features of *Brucella* promoters. Therefore, we examined the *sodC* upstream region for the consensus −10 sequence features known for *E. coli*; in *E. coli* σ^70^- and σ^s^-dependent promoters contain similar −10 core sequences [30]. We could identify two potential −10 sequences, TTTAAT and TATT(T/G)T, upstream to the identified mRNA 5′ end(s). Our reasoning for mutating the targeted nucleotides was based on the assumption that the ‘A’ insertion in *B. neotomae* changed the potential −10 sequence from the optimal TATT(G/T)T to weaker TAATTG. Though our experimental results with the mutated *B. neotomae* and *B. suis* biovar 4 promoters appear to support this hypothesis, we cannot rule out the possibility that the targeted nucleotides could be outside the −10 region but still be critical for the promoter function. Even if our assumption about the effect on the −10 region is not true, the β-galactosidase expression pattern under the mutated promoters (Figs. 7 and 8) indicates that the nucleotides in the targeted region are important for the *Brucella sodC* promoter function. Recently, genomes of several *Brucella* isolates have been sequenced at Broad Institute, Cambridge, MA. Search of these (available at http://www.broad.mit.edu/annotation/genome/brucella_group/MultiHome.html) and other *Brucella* genome sequences present in the databases at GenBank indicates that all *B. abortus* and *B. melitensis* biovars/strains sequenced so far have identical nucleotide sequences in the *sodC* upstream region, whereas *B. canis* and *B. suis* biovars 1, 3 and 4 share 100% sequence identity in this region. However, *B. ovis*, *B. suis* biovar 5 (strain 513, isolated from a rodent), *Brucella* sp. 83/13 (isolated from a rodent) and all marine mammal isolates (*B. pinnipedalis* and *B. ceti*) also contain the same ‘A’ insertion in the *sodC* upstream region as reported here for that of *B. neotomae* and *B. suis* biovar 2, suggesting that the promoter activity in these *Brucella* could similarly be at a lower level.

To the best of our knowledge, there are no published studies documenting the growth characteristics of *B. neotomae* in J774A.1 cells and in tissues of BALB/c and C57BL/6 mice, some of the in vitro and in vivo model systems widely used in *Brucella* research. Our studies show that *B. neotomae* is able to enter and replicate in J774A.1 cells similar to other *Brucella spp.* [25]. However, in BALB/c mice, *B. neotomae* exhibited an interesting growth and persistence kinetics, where it can establish an infection in spleen and liver, replicate until day 7 to increase the bacterial burden by up to 1.8 log, and then gradually decline in numbers. In C57BL/6 mice, *B. neotomae* did not replicate to a level to increase its numbers in livers and spleens, but maintained the similar bacterial load from day 1 to day 7 and then gradually declined. These findings suggest that *B. neotomae* can establish an acute infection in mice but it is unable to maintain a chronic infection like other known virulent *Brucella spp*. This characteristic feature makes *B. neotomae* an attractive candidate to be included in studies to understand the mechanisms employed by virulent *Brucella spp.* in causing chronic infections in mouse models. It should be mentioned that the pBB4SOD plasmid was stably maintained in *B. neotomae*, as ascertained by the similar number of CFU obtained by plating the tissue homogenates containing the recombinant bacteria on TSA and TSA with ampicillin.

Previous studies with *B. abortus* showed that SOD plays a role in establishing and maintaining a chronic infection in mice [11], [12]. However, if reduction in the level of SOD expression by *Brucella spp.* affects their ability to cause chronic infection is not known. Increasing the level of SOD expression in *B. neotomae* did not alter the bacterial survival in BALB/c and C57BL/6 mice (Fig. 10). It is possible that the low level of SOD expression in *B. neotomae* is sufficient to inactivate the host-derived superoxide radicals and any additional increase in the amount of SOD has no functional effect. It is also possible that increasing the level of SOD expression per se is insufficient to compensate for other critical genetic deficiencies of *B. neotomae* affecting its ability to maintain a chronic infection.

In conclusion, we have identified the presence of a single-nucleotide insertion in the promoter region as the cause for the reduced activity of *sodC* promoter of *B. neotomae*. Though promoter nucleotide polymorphism has been shown to modulate gene expression in other bacteria [31], [32], this is the first report demonstrating the occurrence of a single-nucleotide difference affecting promoter function and gene expression in *Brucella spp*. This finding highlights the possibility of single nucleotide polymorphisms in promoter regions contributing to the differences in expression of certain genes among *Brucella* species/biovars/strains.

## References

[1] Corbel MJ (1997). Brucellosis: An overview.. Emerg Infect Dis.

[2] Cutler SJ, Whatmore AM, Commander NJ (2005). Brucellosis - new aspects of an old disease.. J Appl Microbiol.

[3] Godfroid J, Cloeckaert A, Liautard JP, Kohler S, Fretin D (2005). From the discovery of the Malta fever's agent to the discovery of a marine mammal reservoir, brucellosis has continuously been a re-emerging zoonosis.. Vet Res.

[4] Foster G, Osterman BS, Godfroid J, Jacques I, Cloeckaert A (2007). *Brucella ceti sp nov* and *Brucella pinnipedialis sp nov* for *Brucella* strains with cetaceans and seals as their preferred hosts.. Int J Syst Evol Microbiol.

[5] Hubalek Z, Scholz HC, Sedlacek I, Melzer F, Sanogo YO (2007). Brucellosis of the common vole (*Microtus arvalis*).. Vector-Borne and Zoonotic Dis.

[6] Scholz HC, Hubalek Z, Sedlacek I, Vergnaud G, Tomaso H (2008). *Brucella microti sp nov.*, isolated from the common vole *Microtus arvalis*.. Int J Syst Evol Microbiol.

[7] Celli J (2006). Surviving inside a macrophage: The many ways of *Brucella*.. Res Microbiol.

[8] Korshunov SS, Imlay JA (2002). A potential role for periplasmic superoxide dismutase in blocking the penetration of external superoxide into the cytosol of Gram-negative bacteria.. Mol Microbiol.

[9] Beck BL, Tabatabai LB, Mayfield JE (1990). A Protein Isolated from *Brucella abortus* Is a Cu-Zn Superoxide-Dismutase.. Biochemistry.

[10] Bricker BJ, Tabatabai LB, Judge BA, Deyoe BL, Mayfield JE (1990). Cloning, Expression, and Occurrence of the *Brucella* Cu-Zn Superoxide-Dismutase.. Infect Immun.

[11] Gee JM, Valderas MW, Kovach ME, Grippe VK, Robertson GT (2005). The *Brucella abortus* Cu,Zn superoxide dismutase is required for optimal resistance to oxidative killing by murine macrophages an wild-type virulence in experimentally infected mice.. Infect Immun.

[12] Tatum FM, Detilleux PG, Sacks JM, Halling SM (1992). Construction of Cu-Zn superoxide dismutase deletion mutants of *Brucella abortus*: analysis of survival in vitro in epithelial and phagocytic cells and in vivo in mice.. Infect Immun.

[13] Stoenner HG, Lackman DB (1957). A new species of *Brucella* isolated from the desert wood rat, *Neotoma lepida* Thomas.. Am J Vet Res.

[14] Beal GA, Lewis RE, McCullough NB, Claflin RM (1959). Experimental Infection of Swine with *Brucella neotomae*.. Am J Vet Res.

[15] Stoenner HG (1963). The behavior of *Brucella neotomae* and *Brucella suis* in reciprocal superinfection experiments in mice and guinea pigs.. Am J Vet Res.

[16] Vemulapalli R, He Y, Boyle SM, Sriranganathan N, Schurig GG (2000). *Brucella abortus* strain RB51 as a vector for heterologous protein expression and induction of specific Th1 type immune responses.. Infect Immun.

[17] McQuiston JR, Schurig GG, Sriranganathan N, Boyle SM (1995). Transformation of *Brucella* species with suicide and broad host-range plasmids.. Methods Mol Biol.

[18] Vemulapalli R, Duncan AJ, Boyle SM, Sriranganathan M, Toth ME (1998). Cloning and sequencing of *yajC* and *secD* homologs of *Brucella abortus* and demonstration of immune responses to YajC in mice vaccinated with *B. abortus* RB51.. Infect Immun.

[19] Vemulapalli R, He Y, Cravero S, Sriranganathan N, Boyle SM (2000). Overexpression of protective antigen as a novel approach to enhance vaccine efficacy of *Brucella abortus* strain RB51.. Infect Immun.

[20] Onate AA, Vemulapalli R, Andrews E, Schurig GG, Boyle S (1999). Vaccination with live Escherichia coli expressing *Brucella abortus* Cu/Zn superoxide dismutase protects mice against virulent *B. abortus*.. Infect Immun.

[21] Stabel TJ, Sha ZG, Mayfield JE (1994). Periplasmic Location of *Brucella abortus* Cu/Zn Superoxide-Dismutase.. Vet Microbiol.

[22] Vemulapalli R, McQuiston JR, Schurig GG, Sriranganathan N, Halling SM (1999). Identification of an IS711 element interrupting the *wboA* gene of *Brucella abortus* vaccine strain RB51 and a PCR assay to distinguish strain RB51 from other *Brucella* species and strains.. Clin Diagn Lab Immun.

[23] Seleem MN, Vemulapalli R, Boyle SM, Schurig GG, Sriranganathan N (2004). Improved expression vector for *Brucella species*.. Biotechniques.

[24] Miller JH (1992). A short course in bacterial genetics: a laboratory manual and handbook for *Escherichia coli* and related bacteria, Cold Spring Harbor Laboratory, New York..

[25] Wise DJ, Sriranganathan N, Boyle SM, Schurig GG, Frank JF (1998). Evaluation of the intracellular growth of various *Brucella* species in J774A.1 and PU5-1.8 macrophage-like cell lines as an in vitro model of assessing attenuation in vivo..

[26] Benov LT, Fridovich I (1994). *Escherichia coli* Expresses a Copper-Containing and Zinc-Containing Superoxide-Dismutase.. J Biol Chem.

[27] Gort AS, Ferber DM, Imlay JA (1999). The regulation and role of the periplasmic copper, zinc superoxide dismutase of *Escherichia coli*.. Mol Microbiol.

[28] Muffler A, Traulsen DD, Fischer D, Lange R, HenggeAronis R (1997). The RNA-binding protein HF-I plays a global regulatory role which is largely, but not exclusively, due to its role in expression of the sigma(s) subunit of RNA polymerase in *Escherichia coli*.. J Bacteriol.

[29] Roop RM, Gee JM, Robertson GT, Richardson JM, Ng WL (2003). *Brucella* stationary-phase gene expression and virulence.. Ann Rev Microbiol.

[30] Hengge-Aronis R (2004). Molecular mechanisms of heat resistance and stress responses in bacteria.. Bulletin of the International Dairy Federation No 392/2004.

[31] Stermann M, Sedlacek L, Maass S, Bange FC (2004). A Promoter Mutation Causes Differential Nitrate Reductase Activity of *Mycobacterium tuberculosis* and *Mycobacterium bovis*.. J Bacteriol.

[32] Gronewold TMA, Kaiser D (2007). Mutations of the act promoter in *Myxococcus xanthus*.. J Bacteriol.

